# Multiple Pre-Treatment miRNAs Levels in Untreated Major Depressive Disorder Patients Predict Early Response to Antidepressants and Interact with Key Pathways

**DOI:** 10.3390/ijms23073873

**Published:** 2022-03-31

**Authors:** Masaki Kato, Haruhiko Ogata, Hidetoshi Tahara, Akira Shimamoto, Yoshiteru Takekita, Yosuke Koshikawa, Keiichiro Nishida, Shinpei Nonen, Koichiro Higasa, Toshihiko Kinoshita

**Affiliations:** 1 Department of Neuropsychiatry, Kansai Medical University, Osaka 573-1191, Japan; ogatahar@takii.kmu.ac.jp (H.O.); takekity@takii.kmu.ac.jp (Y.T.); koshikay@takii.kmu.ac.jp (Y.K.); nishidak@takii.kmu.ac.jp (K.N.); kinoshit@takii.kmu.ac.jp (T.K.); 2 Department of Cellular and Molecular Biology, Graduate School of Biomedical & Health Sciences, Hiroshima University, Hiroshima 734-8533, Japan; toshi@hiroshima-u.ac.jp; 3 Faculty of Pharmaceutical Sciences, Sanyo-Onoda City University, Sanyo Onoda 756-0084, Japan; shim@rs.socu.ac.jp; 4 Department of Pharmacy, Hyogo University of Health Sciences, Kobe 650-8530, Japan; nonen@huhs.ac.jp; 5 Department of Genome Analysis, Institute of Biomedical Science, Kansai Medical University, Osaka 573-1191, Japan; higasako@hirakata.kmu.ac.jp

**Keywords:** miRNA, miR-483-5p, miR-1202, major depressive disorder, predictor, randomized controlled trial, mirtazapine, selective serotonin reuptake inhibitor, TGF-β, glutamatergic synapse

## Abstract

Major depressive disorder (MDD) is a life-impairing disorder, and early successful treatment is important for a favorable prognosis. However, early response to antidepressants differs widely among individuals, and is difficult to predict pre-treatment. As miRNAs have been reported to play important roles in depression, identification of miRNAs associated with antidepressant treatment responses and their interacting genes and pathways will be beneficial in understanding the predictors and molecular mechanisms of depression treatment. This randomized control trial examined miRNAs correlated with the early therapeutic effect of selective serotonin reuptake inhibitors (SSRIs; paroxetine or sertraline) and mirtazapine monotherapy. Before medication, we comprehensively analyzed the miRNA expression of 92 depressed participants and identified genes and pathways interacting with miRNAs. A total of 228 miRNAs were significantly correlated with depressive symptoms improvements after 2 weeks of SSRIs treatment, with miR-483.5p showing the most robust correlation. These miRNAs are involved in 21 pathways, including TGF-β, glutamatergic synapse, long-term depression, and the mitogen-activated protein kinase (MAPK) signaling pathways. Using these miRNAs enabled us to predict SSRI response at week 2 with a 57% difference. This study shows that pre-treatment levels of miRNAs could be used to predict early responses to antidepressant administration, a knowledge of genes, and an identification of genes and pathways associated with the antidepressant response.

## 1. Introduction

Approximately 5% of the world’s population will experience a depressive episode during a 12-month period [1]. The lifetime prevalence of major depressive disorder (MDD) in adults has been reported by retrospective studies to be 10.6% on average. A much higher prevalence (30–40%) has been indicated by prospective studies [2,3,4]. The prevalence of MDD is more than twice as high in chronic physical illnesses than that in the general population [5,6,7]. According to the World Health Organization, MDD is among the most important causes of disability, accounting for approximately one-fifth of the years lived with disability among adults aged 15 years and over. Moreover, it is a source of increased mortality [8]. Although it is possible to prescribe antidepressants, which have been shown to be more useful for depressed patients than placebos, the rate of remission in response to the initially prescribed antidepressants is still not high [9,10]. In psychiatric practice, it is common to prescribe medications empirically and subjectively. However, there is some trial and error involved in identifying the most effective medication. MDD is a chronic, often relapsing disease [11,12]. This prolonged illness increases the risk of a variety of chronic physical disorders [5,13,14,15]. Therefore, successful treatment leading to early response is important for a favorable prognosis [16,17,18,19]. Mirtazapine and SSRIs are both first-line medications for major depressive disorder and their combination is also suggested for patients who are resistant to the first antidepressant medication used [10,20,21]. In this regard, we have confirmed that mirtazapine (MIR) is superior to selective serotonin reuptake inhibitors (SSRIs) in treatment response as early as 2 weeks after the initiation of treatment. There was no significant difference between the two types of medication after one month of treatment [10], which is consistent with the findings of a previous meta-analysis [22]. The pharmacological profile of mirtazapine, an antagonist of histamine H1, serotonin 2A/2C, alpha 2 adrenergic receptors, and SSRIs that bind to the serotonin transporter, may explain these clinical differences [23,24]. However, monoamine neurotransmission is complex and involves several neurotransmitters, including pre- and post-synaptic receptors, transporters, and enzymes that determine the availability and effects of specific monoamine neurotransmitters [25,26,27,28]. In addition, downstream changes in signaling pathways, such as changes in gene expression, neuroplasticity, immune, and inflammation-related pathways, may play an essential role [29,30,31]. The mechanisms by which antidepressants exert their effects are still not completely understood, and various hypotheses are being investigated to elucidate it [25,32,33,34,35,36].

MicroRNAs (miRNAs) are a class of small non-coding endogenous RNAs of between 18 and 25 nucleotides in length. They play roles in the regulation of gene expression at the post-translational level [37] and are involved in a range of biological processes associated with the central nervous system, including neural plasticity, neurogenesis, and stress-related responses [38,39]. Accumulating evidence indicates that the dysregulation of miRNAs in peripheral blood and brain tissue may be associated with depression [40,41,42,43,44] and suicide [41,45]; however, only a few studies have investigated the role miRNAs as biomarkers involved in responses to antidepressants [40,41,42,43,44,45,46,47,48,49,50]. Among these, only two studies have evaluated the potential utility of pre-treatment miRNA levels in predicting treatment responses to antidepressants, even using a candidate approach [46,50]; none have used a high-throughput approach.

An early response and remission are desirable treatment goals for depressed patients. However, there are individual differences in the responses to both SSRIs and mirtazapine; some individuals show an early favorable response, while others do not. It is difficult to predict such individual differences in early improvement with each of these classes of drugs; thus, the identification of objective predictors is desirable. As the findings of the aforementioned studies have indicated, the expression levels of miRNAs associated with depression pathology and treatment response differ among individuals, and thus predicting the effects of antidepressants based on miRNA expression patterns may be clinically useful. In this study, we analyzed the correlation between pre-treatment plasma miRNA levels and the therapeutic effects of antidepressants in untreated MDD patients and sought to identify specific miRNAs that contribute to the early therapeutic response to MIR or SSRIs, as well as genes and biological pathways in which these miRNAs are involved.

## 2. Results

### 2.1. Baseline Analysis

Among the MDD outpatients randomized to receive MIR or SSRIs in Step I of the GUNDAM study [10], data on plasma miRNA levels were obtained from 92 participants at the study baseline prior to the administration of medication. After undertaking quality control, 78 (MIR 40/SSRI 38; paroxetine (PAX) 21 or sertraline (SER) 17) participants were included in the subsequent analysis. Table 1 presents the descriptive characteristics of the 78 study subjects. With the exception of the duration of the current depressive episode, there were no significant differences among the patients assigned to the different therapeutic agent groups. The average doses of PAX, SER, and MIR administered during weeks 2 and 4 were 23.7 and 35.5 mg/day, 75.0 and 86.8 mg/day, and 26.8 and 32.8 mg/day, respectively, and the changes in HAM-D score from the study baseline for MIR and SSRIs were −6.8 and −5.3 at week 2, and −9.8 and −9.6 at week 4, respectively. Among the participants, 19.2% (n = 15; MIR 7/SSRI 8) showed responses 2 weeks after the initiation of treatment, whereas after 4 weeks of treatment, 50.0% (n = 39; MIR 17/SSRI 22) of the subjects showed responses. None of the assessed miRNAs showed an association with baseline severity, as assessed by the HAM-D score, whereas the baseline HAM-D score was correlated with the HAM-D score change at weeks 2 and 4.

### 2.2. Associations between miRNA and Treatment Response

#### 2.2.1. SSRIs

**Figure 1:**
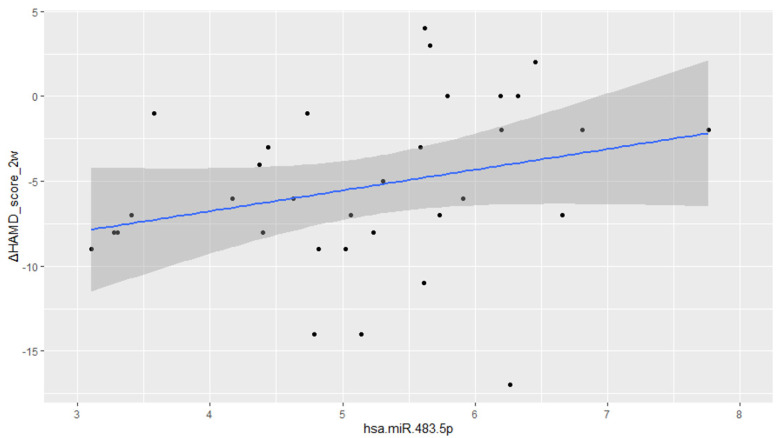
Correlations and regression curves between the miR-483-5p and Hamilton Rating Scale for Depression score changes at week 2.

In patients administered SSRIs, the plasma levels of 228 miRNAs were observed to be significantly correlated with changes in HAM-D score at week 2, after FDR correction for multiple statistical tests (Supplementary Table S1), all of which were inversely correlated with an improvement in HAM-D score. The coefficient of normalized miRNAs for the change in HAM-D score over 2 weeks was 4.27 (maximum) and 1.34 (minimum). Among these miRNAs, miR-483.5p (coefficient = 2.50, P = 7.90 × 10^−5^, F = 8.26, R^2^ = 0.59; Figure 1) and miR-3151.5p (coefficient = 3.51, P = 0.0001, F = 8.95, R^2^ = 0.61) showed the highest and second-highest correlations, respectively, and were also significantly associated with HAM-D score changes at week 4. In addition, a further 23 miRNAs showed a significant association with responses at week 2. However, apart from the HAM-D score change at week 2, the significance of the association with outcomes disappeared following FDR correction.

#### 2.2.2. Mirtazapine

Among patients receiving MIR, the plasma levels of nine miRNAs were found to be significantly correlated with the change in HAM-D score at week 2, of which, two were inversely correlated with an improvement in HAM-D score, whereas the remaining seven were positively correlated. The miR-483-3p showed the most robust positive correlation (*p* = 0.031). The miR-451a was also significantly associated with the response at week 2, whereas miR-483-3p, miR-6893-3p, and miR-4740-3p were significantly associated with the HAM-D score change at week 4. In each case, however, the significance of the association disappeared following FDR correction.

### 2.3. miRNA Target Prediction and Pathway Analysis

**Figure 2:**
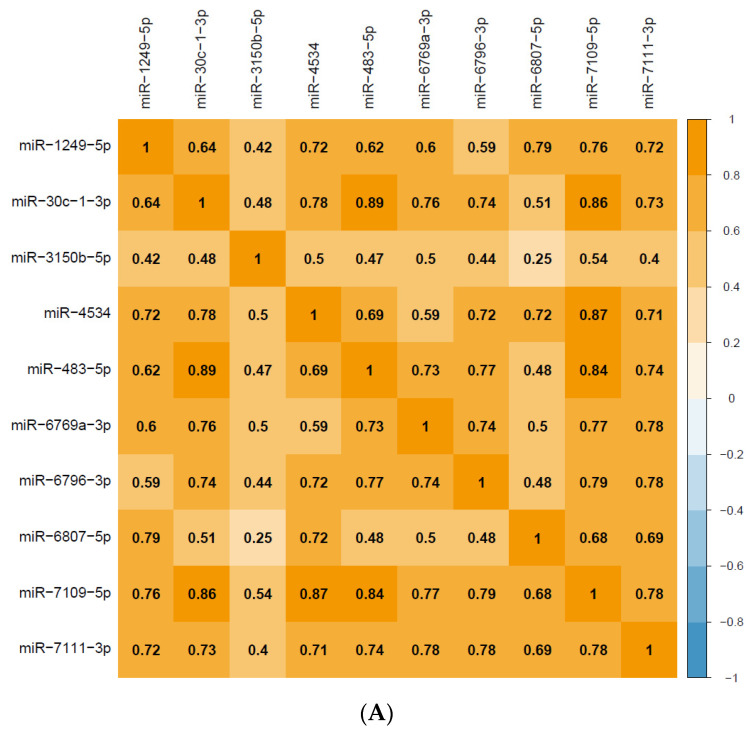

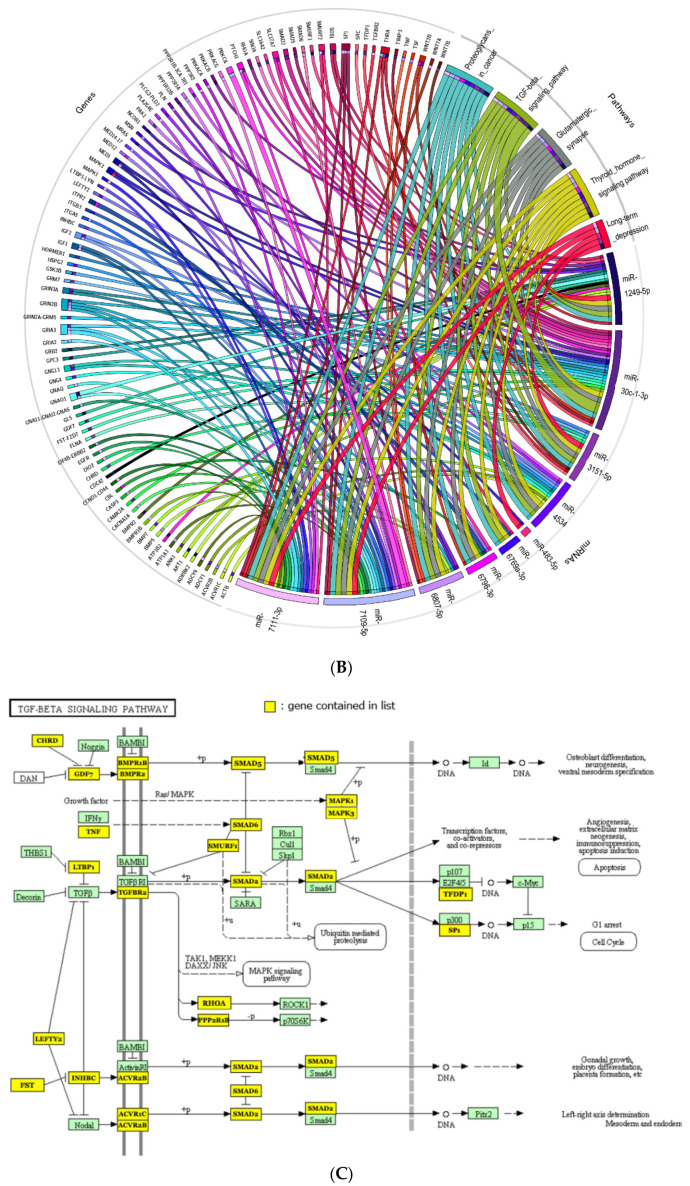

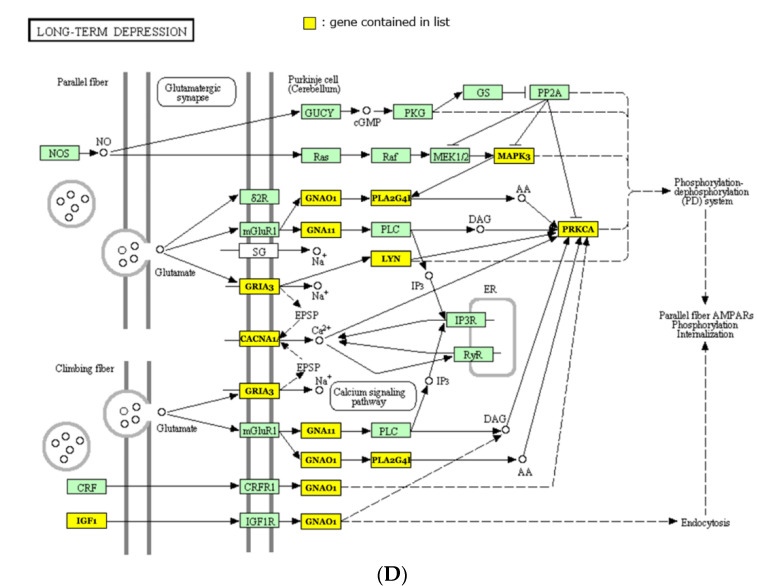
(**A**) A correlation matrix between pre-treatment miRNAs in major depressive disorder patients that were strongly associated with Hamilton Rating Scale for Depression (HAM-D) score reductions after 2 weeks of selective serotonin reuptake inhibitor (SSRI) treatment. Caption: The numbers indicate the correlation coefficients. (**B**) Circos plot of the top 10 miRNAs associated with HAM-D score reduction after 2 weeks of SSRI treatment and the interacting genes and pathways. Caption: After conservative stats based on the probability of jackknifing tests and FDR correction, of 21 pathways that were significantly associated with these 10 miRNAs(miR-483-5p, miR-3151-5p, miR-7109-5p, miR-6807-5p, miR-30c-1-3p, miR-6769a-3p, miR-7111-3p, miR-6796-3p, miR-1249-5p, miR-4534), the five pathways (TGF-β signaling pathway, Proteoglycans in cancer, Long-term depression, Glutamatergic synapse, and Thyroid hormone signaling pathway) and 95 genes that showed the strongest association (adjusted *p* < 0.01) are shown. Each ribbon connects an miRNA with predicted target genes and pathways. The width of the ribbon is proportional to the number of results indicating the interaction. (**C**) Modified “TGF-beta signaling pathway” from KEGG. Caption: The genes in the yellow squares are statistically robust interactions with the top 10 miRNAs associated with HAM-D score reduction after 2 weeks of SSRI treatment. (**D**) Modified “Long-term depression” from KEGG. Caption: The genes in the yellow squares are statistically robust interactions with the top 10 miRNAs associated with HAM-D score reduction after 2 weeks of SSRI treatment.

The top 10 miRNAs (miR-483-5p, miR-3151-5p, miR-7109-5p, miR-6807-5p, miR-30c-1-3p, miR-6769a-3p, miR-7111-3p, miR-6796-3p, miR-1249-5p, and miR-4534), which were positively intercorrelated (Figure 2A) and strongly associated with a HAM-D score reduction after 2 weeks of SSRI treatment, even after FDR correction, were used to identify target genes and associated biological pathways based on in silico analyses of three databases (micro-T-CDS, Tarbase, and TagetScan) using DIANA: miRPath v.3 software (University of Thessaly and Information Management Systems Institute(IMSI), Greece). After conservative analysis based on the probability of a jackknifing test and FDR correction, we identified 21 pathways (Table 2; micro-T-CDS, 20; Tarbase, 1; TagetScan, 1) that were significantly associated with these 10 miRNAs (Figure 2B). The pathways that interacted robustly (adjusted *p* < 0.01) with these 10 miRNAs were as follows: TGF-β signaling pathway, Proteoglycans in cancer, Long-term depression, Glutamatergic synapse, and Thyroid hormone signaling pathway (Table 2). Among these, the TGF-β signaling pathway was identified from two or more databases. A network of these five pathways and 95 genes that showed a strong interaction with these 10 miRNAs is presented in Figure 2B. Figure 2C,D shows the genes interacting with these miRNAs in the TGF-β signaling pathway and long-term depressive pathway, respectively, which are strongly associated with these miRNAs and are also involved in depression.

### 2.4. Heatmap Analysis

**Figure 3:**
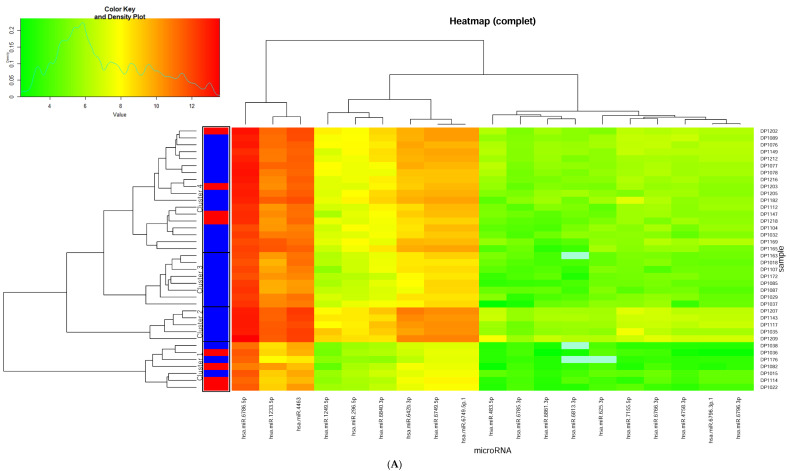

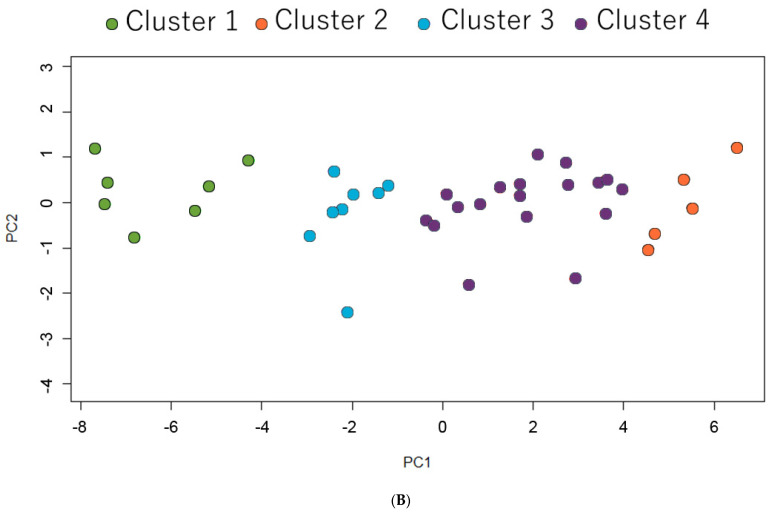
(**A**) Hierarchical clustering of subjects treated with selective serotonin reuptake inhibitors based on pre-treatment miRNA levels. Caption: The heat map was generated using 17 miRNAs that were significantly associated with a Hamilton Rating Scale for Depression score change at week 2 (adjusted *p* < 0.05) and also correlated with week 2 response with an unadjusted *p*-value < 0.01. (**B**) Principal component analysis of subjects classified into four categories based on partitional clustering analysis. Caption: Cluster plot points according to the first two principal components (PC1 and PC2) that explain 91.4% of the total variance.

As an exploratory approach for clinical use, we performed heat map analysis using 17 miRNAs that showed a statistically significant association with HAM-D score change at week 2 (adjusted *p* < 0.05) and were also correlated with the week 2 response with an unadjusted *p*-value < 0.01 (Figure 3A). To extract meaningful clusters, we performed graphical exploration and principal component analysis (PCA), which revealed that subjects could be classified into four clusters (Figure 3B). The percentages of week 2 responses in cluster 1, cluster 2 and cluster 3, and cluster 4 were 57.1%, 0.0%, and 22.2%, respectively. These were higher in cluster 1 and lower in clusters 2 and 3 compared with the average percentage of 21.1%. The results indicate that these miRNAs can be used to predict the group with the highest and lowest likelihood of response at 2 weeks with a significant difference of 57%.

## 3. Discussion

In our randomized control trial, we identified 228 miRNAs that were strongly correlated with an early response to SSRIs, among which, miR-483.5p was characterized by the most robust correlation. Prior to treatment, the levels of these miRNAs were inversely correlated with an improvement in depressive symptoms. We established that these miRNAs are involved in 21 pathways, including TGF-β, glutamatergic synapse and long-term depression, which are known to be associated with MDD. On the basis of an exploratory hierarchical cluster analysis using a selection of these miRNAs, we were able to predict the response at week 2 with a 57% difference. Notably, in the case of patients receiving MIR, having performed correction for multiple testing, we were unable to identify any miRNAs correlated with an early response. Among the top hit miRNAs that showed significant pre-correction associations, miRNA levels were not only inversely correlated with the improvement in depressive symptoms but also were more often positively correlated.

In terms of pathways that were found to interact with the top 10 most significantly associated miRNAs in terms of an early response to SSRIs, the TGF-β signaling pathway was the only one that was identified in more than one database. Of these top 10 miRNAs, eight (miR-3151-5p, miR-6807-5p, miR-30c-1-3p, miR-6769a-3p, miR-7111-3p, miR-6796-3p, miR-1249-5p, and miR-4534) were shown to be associated with this pathway. The genes established to interact with these miRNAs are as follows: transforming growth factor beta receptor 2 gene (TGFBR2); tumor necrosis factor gene (TNF); activin a receptor type 1C (ACVR1C), and 2B genes (ACVR2B); bone morphogenetic protein (BMP)-related genes such as BMP5, BMP7, and BMP receptor type 1B BMPR1B and 2 BMPR2; MAPK1 and MAPK3; SMAD family-related genes such as SMAD family member 2 (SMAD2), 5 (SMAD5) and 6 (SMAD6), SMAD-specific E3 ubiquitin protein ligase 1 (SMURF1), and 2 genes (SMURF2); latent transforming growth factor beta binding protein 1 gene (LTBP1); left-right determination factor 2 gene (LEFTY2), protein phosphatase 2 scaffold subunit alpha gene (PPP2R1A), and beta gene (PPP2R1B); Inhibin subunit beta C (INHBC); chordin (CHRD); Sp1 transcription factor (SP1); growth differentiation factor 7 (GDF7); follistatin (FST); Ras homolog family member A (RHOA); and Transcription factor Dp-1 gene (TFDP1).

Previous studies that focused on predicting the treatment responses to antidepressants using pre-treatment miRNAs have only reported using a candidate factor approach rather than a genome-wide approach. They have indicated that blood miR1202 in pre-treatment depressed patients was inversely correlated with the treatment response to antidepressants [46,50]. This is consistent with our findings in the present study, in which we established that miR1202 was inversely correlated with 2-week HAM-D score improvement and response at week 2 in patients administered SSRIs (Supplementary Table S1). These previous studies have also shown that miR-1202 is correlated with the expression of the gene encoding metabotropic glutamate receptor-4 (GRM4). Although miR-1202 was not included in the interacting gene–pathway analysis in the present study, we did, nevertheless, detect significant associations among the top10 miRNAs and other glutamate-related genes, such as GRM5, 7, GIN2B, GRIA3, GNAO1, HOMER1, and the Glutamatergic synapse pathway. Although the aforementioned two studies assessed the correlation with 8-week remission, whereas we focused on early responses, the partial responses at 2 weeks have, nonetheless, been shown to be strongly correlated with subsequent remission [19,51]. Thus, it would be reasonable to assume that there is a common pathway associated with the treatment response at 2 and 8 weeks. Further interesting studies on miR-1202, including molecular imaging studies, have shown that changes in miR-1202 levels during antidepressant treatment are correlated with changes in brain activity [52], and that genetic variants of the GRM4 gene at the binding site of miR-1202 are potentially associated with a heightened risk of developing depression. Accordingly, these observations would tend to indicate that miR-1202 and constituents of the interacting pathways are among the key molecules involved in the pathogenesis and treatment of depression [53].

In contrast to SSRIs, although we found that no miRNAs associated with the early response to MIR remained significant following FDR correction, the top hit miRNA, namely, miR483-3p, derived from the same dsRNA as the SSRI top hit miRNA, miR483-5p. Of the two strands of RNA duplexes, one miRNA strand is incorporated into the argonaut (Ago) protein as the guide strand during the formation of RNA-induced silencing complexes (RISCs) to become the mature miRNA, whereas the other strand is discarded. Although numerous miRNA precursors have been established to produce a single dominant mature miRNA species, in some cases, both strands function as mature miRNAs [54]. It has been speculated that the mechanisms determining which of the two strands functions mature miRNAs, or whether both strands contribute to this regard, involve control by nucleotide bases on the 5′ side and thermodynamic stability [55]. We found that the plasma levels of miR483-5p were inversely correlated with early improvements in patients receiving SSRIs, whereas the levels of miR483-3p were positively correlated with early improvement in those administered MIR. These findings may thus indicate that the factors determining whether miR483-3p or 5p functions on the maturation of double-stranded miRNAs may influence the early response to SSRI and MIR treatment. In a previous study conducted by Yoshino et al. using the postmortem brains of depressed patients to examine the associations of 333 miRNAs, the authors found miR-483-5p found to be significantly more down-regulated in a synaptic fraction from the dorsolateral prefrontal cortex (dlPFC) of MDD subjects compared with healthy controls [43]. It is thus interesting to note that in the present study, we demonstrated that miR-483-5p, which is dysregulated in the postmortem brains and reduced in synapses of depressed patients, is associated with a long-term depression pathway (Figure 1), along with miR-3151-5p, miR-7109-5p, miR-7111-3p, and miR-1249-5p. This pathway comprises glutaminergic synapse-related genes, and we established that MAPK3, IGF1, GRIA3, CACNA1, GNA11, GNAO1, PLA2G4E, and PRKCA interact with SSRI early response-related miRNAs. Among the 333 miRNAs examined by Yoshino et al., there were seven associated with synapses in the postmortem brains of depressed patients that were not included among the 684 miRNAs that met the analysis criteria specified in the present study. In addition, there are reports on miR-483-5p indicating associations with 483-3p, the IGF2 gene, cancer, and adipogenesis in physical diseases [56,57,58]. miR-3151-5p, which we identified as showing the second-most robust correlation with an early response to SSRIs, has been reported to be associated with certain cancers and leukemia, although to date has not been shown to be associated with psychiatric disorders. In a study by Lopez et al. [59], two of the miRNAs that showed significant changes in response to duloxetine treatment were included in the analyses performed in the present study, of which, miR-6511a-3p did not appear to be associated with a treatment response, whereas the second, miR-2110, was observed to be highly correlated with an early response with a statistically significant adjusted *p*-value. Lopez et al. concluded that the pathway associated with an antidepressant response in depressed patients is the MAPK signaling pathway, which we also identified in the present study. On the basis of the foregoing findings, it appears that the types of miRNAs available for analysis differ depending on race and analytical method. However, given that miRNAs are associated with genes and molecular pathways, not only in isolation but also in correlation with multiple other miRNAs, it is important to both reconfirm the associations of single miRNAs reported in previous studies. An attempt to elucidate common molecular mechanisms underlying the associations of the genes and pathways identified to date.

However, despite the interesting findings of the present study, we must acknowledge that there were several general limitations with respect to the study design. Firstly, although the results obtained in this study were based on rigorous statistical analyses, including FDR correction, our findings are to a certain extent limited by the small cohort size, and accordingly would require validation using larger cohort of MDD patients to enable a better evaluation of the involvement and specificity of the putative miRNAs and pathways. Secondly, we did not include placebo and/or non-treatment groups in the analysis. Although the study was conducted based on a randomized controlled trial design, which can reduce unknown and known biases, the use of placebo and non-treatment groups serve as an effective strategy for distinguishing among responses to antidepressants, placebos, and a spontaneous improvement in symptoms. Thirdly, given that we focused on an early response to antidepressants, we assessed the treatment response after 2 weeks based on pre-treatment miRNA levels as the main outcome, and after 4 weeks as a secondary outcome. In this regard, although a treatment response at 2 weeks is known to contribute significantly to the subsequent course of treatment [19,51], our findings cannot be used to predict the long-term course of treatment, which is important in MDD.

Nevertheless, one of the strengths of this study is that we used a randomized controlled trial design, and consequently there was no drug selection bias. We are thus confident that the influence of known patient characteristics and unknown events was minimized. In addition, the study design enabled us to follow patients who are drug-free and have no history of treatment-resistant depression with a controlled intervention. Furthermore, the use of a comprehensive genome-wide approach and conservative statistical methods, rather than a hypothesis-based candidate approach for the targeted miRNAs, enabled us to analyze not only known but also unknown miRNAs.

## 4. Methods

### 4.1. Study Design and Participants

This study is part of the Step I portion of the Genotype Utility Needed for Depression Antidepressant Medication (GUNDAM) study, which is a project focused on personalized first-line treatment and subsequent combination treatment based on a series of biological and clinical factors. The methods and designs of the GUNDAM study have previously been described elsewhere [10]. Briefly, GUNDAM is a two-step, open-label, randomized, flexible-dose, 8-week study, in Step I (4 weeks) of which, the responses of drug-free MDD patients to treatment with MIR and SSRIs were evaluated. The study was registered in the UMIN (University Hospital Medical Information; No. 000006417). The subjects included were 20- to 75-year-old outpatients, who met the criteria of diagnosis of MDD according to the Structured Clinical Interview for Diagnostic and Statistical Manual of Mental Disorders, Fourth Edition Axis I Disorders [60], Japanese, scoring at least 14 in the 17-item Hamilton Rating Scale for Depression (HAM-D 17) [61]. They had been free of psychotropic drugs for at least 14 days prior to entering into the study. Subjects with clinically significant unstable medical illness, pregnancy, a principal psychiatric diagnosis other than MDD, a history of substance abuse or dependence active within the previous 6 months, or a history of treatment-resistant depression, defined as non-response to two or more antidepressants and electroconvulsive therapy within the previous 6 months, were excluded. The diagnoses were performed by two independent senior psychiatrists and confirmed by a third.

Participants were randomly assigned to an MIR or SSRI (PAX or SER) group. The initial doses for MIR, PAX, and SER were 15, 10, and 25 mg/day, respectively. If there were no problems regarding tolerability, these doses were increased to 30, 20, and 50 mg/day within 2 weeks, and up to 45, 40, and 100 mg/day within 4 weeks, respectively. All patients were evaluated at baseline and bi-weekly thereafter until the end of the study using the HAM-D 17 scale. The rate of response was defined as at least a 50% reduction from the baseline score.

### 4.2. Microarray Analysis of miRNA Expression

Human plasma samples were obtained from pre-treatment patients with MDD. Total RNA was extracted from 300 µL serum samples using a 3D-Gene RNA extraction reagent provided with a liquid sample kit (Toray Industries, Inc., Kanagawa, Japan). Comprehensive miRNA expression analysis was performed using a 3D-Gene miRNA Labeling kit and a 3D-Gene Human miRNA Oligo Chip (Toray Industries, Inc., Kanagawa, Japan), which was designed to detect 2555 miRNA sequences registered in the miRBase release 20 database (http://www.mirbase.org/) (accessed on 02 March 2022). When an miRNA was detected in only a proportion of the subjects (less than 80%), the assay of the miRNA was regarded as unreliable. Of the 2555 miRNAs assessed, data obtained for 684 were considered sufficiently reliable and used for subsequent statistical analysis, having initially undergone quantile normalization. Signals that were less than the average background signal value ±2 SD or considered abnormal in the scanned images and thus unsuitable as data were removed as missing areas.

### 4.3. Statistical Analyses

The change in HAM-D score at week 2 was used as the primary outcome, and that at week 4 and the response rates at weeks 2 and 4 were used as secondary outcomes. Multiple regression models for continuous variables and logistic regression models for binary outcomes were fitted to estimate the effects of treatment on the outcomes. Results with an alpha level lower than 0.05 were considered significant. The models were controlled for age, sex, duration of current depressive episode, and pre-treatment HAN-D score. The Benjamini–Hochberg false discovery rate (FDR) correction was used to correct for the proportion of miRNAs likely to be identified as significant by chance by multiple tests (adjusted *p*-value of 0.05). All analyses were performed using the R Statistics Package v. 3.51 (R Foundation for Statistical Computing, http://www.R-project.org) (accessed on 2 March 2022). To validate our miRNA target prediction and pathway analyses, we performed in silico analysis using DIANA: miRPath v.3, which facilitates identification of common miRNA targets using DAVID ease scores with Conservative Stats based on jackknifing test probability and the Benjamini–Hochberg FDR correction derived from DIANA microT-CDS (v5.0), TarBase v7.0 (University of Thessaly and Information Management Systems Institute (IMSI), Greece), and the Target scan database. For pathway analysis, we used the top 10 miRNAs, even if more than 10 were significant, based on previous experience indicating that the use of an excessive number of miRNAs can lead to difficulties in interpreting the results [62]. To generate heat maps, we performed hierarchical cluster analysis with complete linkage to identify patient clusters based on baseline plasma miRNA levels. The Euclidean distance was used to measure the dissimilarity between each pair of observations, under a hierarchical clustering approach for identifying clusters of patients.

## 5. Conclusions

We found that the pre-treatment levels of 228 miRNAs were associated with an early response to selective serotonin reuptake inhibitors. The most strongly correlated of these, miR483-5p, has previously been reported to be dysregulated in the postmortem brains of depressed patients and reduced at the synapses therein [43]. In addition, we found that miR-1202, which has consistently been reported to be associated with treatment response to antidepressants using the candidate approach [46,50], was also correlated with treatment response based on our genome-wide approach. In silico pathway analysis enabled us to identify 21 pathways associated with the 228 miRNAs and their interacting genes such as TGFBR2, TNF, MAPK, SMAD, including the TGF-β, glutamatergic synapse, and long-term depression pathways, associated with major depressive disorder, as well as the MAPK signaling pathway reported in previous studies [30,59,63,64,65,66,67,68]. Although preliminary, the findings of this study indicate that these miRNAs could be used to predict groups with the highest and least likelihood of antidepressant response at 2 weeks. That is, the response rate after 2 weeks of SSRI treatment is 21% in the usual treatment, but by evaluating pre-treatment miRNAs, it is possible to divide the group into two groups: one that can expect a 57% response and the other that can expect almost no response (0%). In contrast to SSRIs, we detected no miRNAs significantly correlated with a mirtazapine treatment response after statistical correction. This may suggest using mirtazapine as a monotherapy or in combination early in treatment for patients who are predicted by miRNAs not to respond early to SSRIs. Alternatively, non-pharmacologic or non-invasive neuromodulation therapies might be considered for such patients [69,70,71]. We believe that the findings of this study could contribute to predicting early responses to antidepressant therapy by using miRNAs from untreated patients, as well as enhance our understanding of the genes and pathways associated with antidepressant response. In future studies, we hope to validate our findings using a larger sample size.

## Figures and Tables

**Table 1:** Baseline clinical characteristics of depressed patients participating in this study.

	Total (n = 78)		Mirtazapine (n = 40)		SSRIs (n = 38)		*p*
	%		%		%		
Sex (female)	48.7%		50.0%		47.3%		n.s.
First episode	68.4%		71.1%		65.8%		n.s.
Family psychiatric history	29.2%		25.7%		32.4%		n.s.
Physical comorbidity	38.7%		35.1%		42.1%		n.s.
Smoking	2.7%		5.4%		0.0%		n.s.
Drinking	22.7%		23.7%		21.6%		n.s.
Occupational status: Employed	79.5%		80.0%		78.9%		n.s.
	Mean	SD	Mean	SD	Mean	SD	
Age	47.7	16.8	48.4	16.4	47	17.4	n.s.
Duration of current MDD episode (months)	8.6	18.1	6	10	11.4	23.7	0.015
HAM-D 17 items total score	21	4.7	21.5	5.1	20.5	4.4	n.s.

SSRIs: selective serotonin reuptake inhibitors, MDD: major depressive disorder, HAM-D: Hamilton depression rating scale.

**Table 2:** Twenty-one pathways showed an interaction with the top 10 miRNAs associated with a Hamilton Rating Scale for Depression score reduction after 2 weeks of SSRI treatment. Caption: The 21 pathways that were significantly associated with 10 miRNAs (miR-483-5p, miR-3151-5p, miR-7109-5p, miR-6807-5p, miR-30c-1-3p, miR-6769a-3p, miR-7111-3p, miR-6796-3p, miR-1249-5p, miR-4534). *p*-values were obtained following conservative stats analysis based on the probability of a jackknifing test and FDR correction.

Pathway	Database	*p*-Value	#Genes	#miRNAs
TGF-beta signaling pathway	microT-CDS/Tarbase	0.006/0.036	21/6	8/1
Proteoglycans in cancer	microT-CDS	<0.001	41	9
Long-term depression	TargetScan	0.002	13	5
Glutamatergic synapse	microT-CDS	0.006	26	8
Thyroid hormone signaling pathway	microT-CDS	0.006	26	9
Amphetamine addiction	microT-CDS	0.024	16	7
Morphine addiction	microT-CDS	0.025	22	8
Endocrine and other factor-regulated calcium reabsorption	microT-CDS	0.027	13	6
Calcium signaling pathway	microT-CDS	0.027	36	8
Hippo signaling pathway	microT-CDS	0.027	28	8
Signaling pathways regulating pluripotency of stem cells	microT-CDS	0.027	27	9
Dilated cardiomyopathy	microT-CDS	0.027	22	9
MAPK signaling pathway	microT-CDS	0.028	49	9
Circadian entrainment	microT-CDS	0.028	24	9
Colorectal cancer	microT-CDS	0.038	13	5
ErbB signaling pathway	microT-CDS	0.038	17	7
ECM-receptor interaction	microT-CDS	0.038	16	7
Axon guidance	microT-CDS	0.038	25	8
Cytokine-cytokine receptor interaction	microT-CDS	0.038	37	9
Endocytosis	microT-CDS	0.038	37	10
Focal adhesion	microT-CDS	0.049	40	10

## Supplementary Materials

The following supporting information can be downloaded at: https://www.mdpi.com/article/10.3390/ijms23073873/s1.

## References

[1] Ferrari A.J., Somerville A.J., Baxter A.J., Norman R., Patten S.B., Vos T., Whiteford H.A. (2013). Global variation in the prevalence and incidence of major depressive disorder: A systematic review of the epidemiological literature. Psychol. Med..

[2] Bromet E.J., Andrade L.H., Bruffaerts R., Williams D.R., Stein D.J., Scott K.M., de Jonge P., Kessler R.C. (2018). Major Depressive Disorder. Mental Disorders Around the World: Facts and Figures from the WHO World Mental Health Surveys.

[3] Moffitt T.E., Caspi A., Taylor A., Kokaua J., Milne B.J., Polanczyk G., Poulton R. (2010). How common are common mental disorders? Evidence that lifetime prevalence rates are doubled by prospective versus retrospective ascertainment. Psychol. Med..

[4] Herrman H., Patel V., Kieling C., Berk M., Buchweitz C., Cuijpers P., Furukawa T.A., Kessler R.C., Kohrt B.A., Maj M. (2022). Time for united action on depression: A Lancet-World Psychiatric Association Commission. Lancet.

[5] Gold S.M., Kohler-Forsberg O., Moss-Morris R., Mehnert A., Miranda J.J., Bullinger M., Steptoe A., Whooley M.A., Otte C. (2020). Comorbid depression in medical diseases. Nat. Rev. Dis. Primers.

[6] Walker J., Burke K., Wanat M., Fisher R., Fielding J., Mulick A., Puntis S., Sharpe J., Esposti M.D., Harriss E. (2018). The prevalence of depression in general hospital inpatients: A systematic review and meta-analysis of interview-based studies. Psychol. Med..

[7] Wang J., Wu X., Lai W., Long E., Zhang X., Li W., Zhu Y., Chen C., Zhong X., Liu Z. (2017). Prevalence of depression and depressive symptoms among outpatients: A systematic review and meta-analysis. BMJ Open.

[8] World Health Organization The Mental Health Gap Action Program (mhGAP). https://www.who.int/publications/i/item/9789241596206:.

[9] Fava M., Detke M.J., Balestrieri M., Wang F., Raskin J., Perahia D. (2006). Management of depression relapse: Re-initiation of duloxetine treatment or dose increase. J. Psychiatry Res..

[10] Kato M., Takekita Y., Koshikawa Y., Sakai S., Bandou H., Nishida K., Sunada N., Onohara A., Hatashita Y., Serretti A. (2017). Non response at week 4 as clinically useful indicator for antidepressant combination in major depressive disorder. A sequential RCT. J. Psychiatry Res..

[11] Kato M., Hori H., Inoue T., Iga J., Iwata M., Inagaki T., Shinohara K., Imai H., Murata A., Mishima K. (2021). Discontinuation of antidepressants after remission with antidepressant medication in major depressive disorder: A systematic review and meta-analysis. Mol. Psychiatry.

[12] Rush A.J., Trivedi M.H., Wisniewski S.R., Nierenberg A.A., Stewart J.W., Warden D., Niederehe G., Thase M.E., Lavori P.W., Lebowitz B.D. (2006). Acute and Longer-Term Outcomes in Depressed Outpatients Requiring One or Several Treatment Steps: A STAR*D Report. Am. J. Psychiatry.

[13] Penninx B.W., Milaneschi Y., Lamers F., Vogelzangs N. (2013). Understanding the somatic consequences of depression: Biological mechanisms and the role of depression symptom profile. BMC Med..

[14] Cohen S., Rodriquez M.S. (1995). Pathways linking affective disturbances and physical disorders. Health Psychol..

[15] Sáiz-Vázquez O., Gracia-García P., Ubillos-Landa S., Puente-Martínez A., Casado-Yusta S., Olaya B., Santabárbara J. (2021). Depression as a Risk Factor for Alzheimer’s Disease: A Systematic Review of Longitudinal Meta-Analyses. J. Clin. Med..

[16] Trivedi M.H., Morris D.W., Wisniewski S.R., Lesser I., Nierenberg A.A., Daly E., Kurian B.T., Gaynes B.N., Balasubramani G.K., Rush A.J. (2013). Increase in work productivity of depressed individuals with improvement in depressive symptom severity. Am J. Psychiatry.

[17] Habert J., Katzman M.A., Oluboka O.J., McIntyre R.S., McIntosh D., MacQueen G.M., Khullar A., Milev R.V., Kjernisted K.D., Chokka P.R. (2016). Functional Recovery in Major Depressive Disorder: Focus on Early Optimized Treatment. Prim. Care Companion CNS Disord..

[18] Kato M., Serretti A., Nonen S., Takekita Y., Wakeno M., Azuma J., Kinoshita T. (2015). Genetic variants in combination with early partial improvement as a clinical utility predictor of treatment outcome in major depressive disorder: The result of two pooled RCTs. Transl. Psychiatry.

[19] Szegedi A., Jansen W.T., van Willigenburg A.P., van der Meulen E., Stassen H.H., Thase M.E. (2009). Early improvement in the first 2 weeks as a predictor of treatment outcome in patients with major depressive disorder: A meta-analysis including 6562 patients. J. Clin. Psychiatry.

[20] Bauer M., Pfennig A., Severus E., Whybrow P.C., Angst J., Moller H.J. (2013). World Federation of Societies of Biological Psychiatry (WFSBP) guidelines for biological treatment of unipolar depressive disorders, part 1: Update 2013 on the acute and continuation treatment of unipolar depressive disorders. World J. Biol. Psychiatry.

[21] Kennedy S.H., Lam R.W., McIntyre R.S., Tourjman S.V., Bhat V., Blier P., Hasnain M., Jollant F., Levitt A.J., MacQueen G.M. (2016). Canadian Network for Mood and Anxiety Treatments (CANMAT) 2016 Clinical Guidelines for the Management of Adults with Major Depressive Disorder: Section 3. Pharmacological Treatments. Can. J. Psychiatry.

[22] Watanabe N., Omori I.M., Nakagawa A., Cipriani A., Barbui C., McGuire H., Churchill R., Furukawa T.A. (2008). Mirtazapine versus other antidepressants in the acute-phase treatment of adults with major depression: Systematic review and meta-analysis. J. Clin. Psychiatry.

[23] Anttila S.A., Leinonen E.V. (2001). A review of the pharmacological and clinical profile of mirtazapine. CNS Drug Rev..

[24] Haddjeri N., Blier P., de Montigny C. (1996). Effect of the alpha-2 adrenoceptor antagonist mirtazapine on the 5-hydroxytryptamine system in the rat brain. J. Pharmacol. Exp. Ther..

[25] Otte C., Gold S.M., Penninx B.W., Pariante C.M., Etkin A., Fava M., Mohr D.C., Schatzberg A.F. (2016). Major depressive disorder. Nat. Rev. Dis. Primers.

[26] Tanaka M., Tóth F., Polyák H., Szabó Á., Mándi Y., Vécsei L. (2021). Immune Influencers in Action: Metabolites and Enzymes of the Tryptophan-Kynurenine Metabolic Pathway. Biomedicines.

[27] Nemeroff C.B. (2020). The State of Our Understanding of the Pathophysiology and Optimal Treatment of Depression: Glass Half Full or Half Empty?. Am. J. Psychiatry.

[28] Sakurai H., Yonezawa K., Tani H., Mimura M., Bauer M., Uchida H. (2022). Novel Antidepressants in the Pipeline (Phase II and III): A Systematic Review of the US Clinical Trials Registry. Pharmacopsychiatry.

[29] Hill A.S., Sahay A., Hen R. (2015). Increasing Adult Hippocampal Neurogenesis is Sufficient to Reduce Anxiety and Depression-Like Behaviors. Neuropsychopharmacology.

[30] Atake K., Hori H., Kageyama Y., Koshikawa Y., Igata R., Tominaga H., Katsuki A., Bando H., Sakai S., Nishida K. (2022). Pretreatment plasma cytokine levels as potential predictors of short-term remission of depression. World J. Biol. Psychiatry.

[31] Sharp T. (2013). Molecular and cellular mechanisms of antidepressant action. Curr. Top Behav. Neurosci..

[32] Pena-Vargas C., Armaiz-Pena G., Castro-Figueroa E. (2021). A Biopsychosocial Approach to Grief, Depression, and the Role of Emotional Regulation. Behav. Sci..

[33] Tanaka M., Vecsei L. (2021). Editorial of Special Issue “Crosstalk between Depression, Anxiety, and Dementia: Comorbidity in Behavioral Neurology and Neuropsychiatry”. Biomedicines.

[34] Sanacora G., Zarate C.A., Krystal J.H., Manji H.K. (2008). Targeting the glutamatergic system to develop novel, improved therapeutics for mood disorders. Nat. Rev. Drug Discov..

[35] Hashimoto K. (2016). Ketamine’s antidepressant action: Beyond NMDA receptor inhibition. Expert Opin. Ther. Targets.

[36] Noto C., Rizzo L.B., Mansur R.B., McIntyre R.S., Maes M., Brietzke E. (2014). Targeting the inflammatory pathway as a therapeutic tool for major depression. Neuroimmunomodulation.

[37] Qureshi I.A., Mehler M.F. (2012). Emerging roles of non-coding RNAs in brain evolution, development, plasticity and disease. Nat. Rev. Neurosci..

[38] Wehrspaun C.C., Ponting C.P., Marques A.C. (2014). Brain-expressed 3′UTR extensions strengthen miRNA cross-talk between ion channel/transporter encoding mRNAs. Front. Genet..

[39] Belzeaux R., Lin C.W., Ding Y., Bergon A., Ibrahim E.C., Turecki G., Tseng G., Sibille E. (2016). Predisposition to treatment response in major depressive episode: A peripheral blood gene coexpression network analysis. J. Psychiatry Res..

[40] Tavakolizadeh J., Roshanaei K., Salmaninejad A., Yari R., Nahand J.S., Sarkarizi H.K., Mousavi S.M., Salarinia R., Rahmati M., Mousavi S.F. (2018). MicroRNAs and exosomes in depression: Potential diagnostic biomarkers. J. Cell Biochem..

[41] Smalheiser N.R., Lugli G., Rizavi H.S., Torvik V.I., Turecki G., Dwivedi Y. (2012). MicroRNA expression is down-regulated and reorganized in prefrontal cortex of depressed suicide subjects. PLoS ONE.

[42] Fan H.M., Sun X.Y., Guo W., Zhong A.F., Niu W., Zhao L., Dai Y.H., Guo Z.M., Zhang L.Y., Lu J. (2014). Differential expression of microRNA in peripheral blood mononuclear cells as specific biomarker for major depressive disorder patients. J. Psychiatry Res..

[43] Yoshino Y., Roy B., Dwivedi Y. (2021). Differential and unique patterns of synaptic miRNA expression in dorsolateral prefrontal cortex of depressed subjects. Neuropsychopharmacology.

[44] Cao D.D., Li L., Chan W.Y. (2016). MicroRNAs: Key Regulators in the Central Nervous System and Their Implication in Neurological Diseases. Int. J. Mol. Sci..

[45] Belzeaux R., Fiori L.M., Lopez J.P., Boucekine M., Boyer L., Blier P., Farzan F., Frey B.N., Giacobbe P., Lam R.W. (2019). Predicting Worsening Suicidal Ideation with Clinical Features and Peripheral Expression of Messenger RNA and MicroRNA during Antidepressant Treatment. J. Clin. Psychiatry.

[46] Lopez J.P., Lim R., Cruceanu C., Crapper L., Fasano C., Labonte B., Maussion G., Yang J.P., Yerko V., Vigneault E. (2014). miR-1202 is a primate-specific and brain-enriched microRNA involved in major depression and antidepressant treatment. Nat. Med..

[47] Belzeaux R., Bergon A., Jeanjean V., Loriod B., Formisano-Treziny C., Verrier L., Loundou A., Baumstarck-Barrau K., Boyer L., Gall V. (2012). Responder and nonresponder patients exhibit different peripheral transcriptional signatures during major depressive episode. Transl. Psychiatry.

[48] Issler O., Haramati S., Paul E.D., Maeno H., Navon I., Zwang R., Gil S., Mayberg H.S., Dunlop B.W., Menke A. (2014). MicroRNA 135 is essential for chronic stress resiliency, antidepressant efficacy, and intact serotonergic activity. Neuron.

[49] He S., Liu X., Jiang K., Peng D., Hong W., Fang Y., Qian Y., Yu S., Li H. (2016). Alterations of microRNA-124 expression in peripheral blood mononuclear cells in pre- and post-treatment patients with major depressive disorder. J. Psychiatry Res..

[50] Fiori L.M., Lopez J.P., Richard-Devantoy S., Berlim M., Chachamovich E., Jollant F., Foster J., Rotzinger S., Kennedy S.H., Turecki G. (2017). Investigation of miR-1202, miR-135a, and miR-16 in Major Depressive Disorder and Antidepressant Response. Int. J. Neuropsychopharmacol..

[51] Wagner S., Engel A., Engelmann J., Herzog D., Dreimuller N., Muller M.B., Tadic A., Lieb K. (2017). Early improvement as a resilience signal predicting later remission to antidepressant treatment in patients with Major Depressive Disorder: Systematic review and meta-analysis. J. Psychiatry Res..

[52] Lopez J.P., Pereira F., Richard-Devantoy S., Berlim M., Chachamovich E., Fiori L.M., Niola P., Turecki G., Jollant F. (2017). Co-Variation of Peripheral Levels of miR-1202 and Brain Activity and Connectivity during Antidepressant Treatment. Neuropsychopharmacology.

[53] Dadkhah T., Rahimi-Aliabadi S., Jamshidi J., Ghaedi H., Taghavi S., Shokraeian P., Akhavan-Niaki H., Tafakhori A., Ohadi M., Darvish H. (2017). A genetic variant in miRNA binding site of glutamate receptor 4, metabotropic (GRM4) is associated with increased risk of major depressive disorder. J. Affect. Disord..

[54] Chiang H.R., Schoenfeld L.W., Ruby J.G., Auyeung V.C., Spies N., Baek D., Johnston W.K., Russ C., Luo S., Babiarz J.E. (2010). Mammalian microRNAs: Experimental evaluation of novel and previously annotated genes. Genes Dev..

[55] Suzuki H.I., Katsura A., Yasuda T., Ueno T., Mano H., Sugimoto K., Miyazono K. (2015). Small-RNA asymmetry is directly driven by mammalian Argonautes. Nat. Struct. Mol. Biol..

[56] Liu M., Roth A., Yu M., Morris R., Bersani F., Rivera M.N., Lu J., Shioda T., Vasudevan S., Ramaswamy S. (2013). The IGF2 intronic miR-483 selectively enhances transcription from IGF2 fetal promoters and enhances tumorigenesis. Genes Dev..

[57] Chen K., He H., Xie Y., Zhao L., Zhao S., Wan X., Yang W., Mo Z. (2015). miR-125a-3p and miR-483-5p promote adipogenesis via suppressing the RhoA/ROCK1/ERK1/2 pathway in multiple symmetric lipomatosis. Sci. Rep..

[58] Pepe F., Visone R., Veronese A. (2018). The Glucose-Regulated MiR-483-3p Influences Key Signaling Pathways in Cancer. Cancers.

[59] Lopez J.P., Fiori L.M., Cruceanu C., Lin R., Labonte B., Cates H.M., Heller E.A., Vialou V., Ku S.M., Gerald C. (2017). MicroRNAs 146a/b-5 and 425-3p and 24-3p are markers of antidepressant response and regulate MAPK/Wnt-system genes. Nat. Commun..

[60] American Psychiatric Association (1994). Diagnostic and Statistical Manual of Mental Disorders.

[61] Hamilton M. (1967). Development of a rating scale for primary depressive illness. Br. J. Soc. Clin. Psychol..

[62] Vlachos I.S., Zagganas K., Paraskevopoulou M.D., Georgakilas G., Karagkouni D., Vergoulis T., Dalamagas T., Hatzigeorgiou A.G. (2015). DIANA-miRPath v3.0: Deciphering microRNA function with experimental support. Nucleic Acids Res..

[63] Caraci F., Spampinato S.F., Morgese M.G., Tascedda F., Salluzzo M.G., Giambirtone M.C., Caruso G., Munafo A., Torrisi S.A., Leggio G.M. (2018). Neurobiological links between depression and AD: The role of TGF-β1 signaling as a new pharmacological target. Pharmacol. Res..

[64] Liput D.J., Puhl H.L., Dong A., He K., Li Y., Lovinger D.M. (2022). 2-Arachidonoylglycerol mobilization following brief synaptic stimulation in the dorsal lateral striatum requires glutamatergic and cholinergic neurotransmission. Neuropharmacology.

[65] Hiew L.F., Poon C.H., You H.Z., Lim L.W. (2021). TGF-β/Smad Signalling in Neurogenesis: Implications for Neuropsychiatric Diseases. Cells.

[66] Krieglstein K., Zheng F., Unsicker K., Alzheimer C. (2011). More than being protective: Functional roles for TGF-β/activin signaling pathways at central synapses. Trends Neurosci..

[67] Behl T., Rana T., Alotaibi G.H., Shamsuzzaman M., Naqvi M., Sehgal A., Singh S., Sharma N., Almoshari Y., Abdellatif A.A.H. (2022). Polyphenols inhibiting MAPK signalling pathway mediated oxidative stress and inflammation in depression. Biomed. Pharmacother..

[68] Wang X.L., Yuan K., Zhang W., Li S.X., Gao G.F., Lu L. (2020). Regulation of Circadian Genes by the MAPK Pathway: Implications for Rapid Antidepressant Action. Neurosci. Bull..

[69] Zhou X., Teng T., Zhang Y., Del Giovane C., Furukawa T.A., Weisz J.R., Li X., Cuijpers P., Coghill D., Xiang Y. (2020). Comparative efficacy and acceptability of antidepressants, psychotherapies, and their combination for acute treatment of children and adolescents with depressive disorder: A systematic review and network meta-analysis. Lancet Psychiatry.

[70] Borgomaneri S., Battaglia S., Sciamanna G., Tortora F., Laricchiuta D. (2021). Memories are not written in stone: Re-writing fear memories by means of non-invasive brain stimulation and optogenetic manipulations. Neurosci. Biobehav. Rev..

[71] McGrath C.L., Kelley M.E., Holtzheimer P.E., Dunlop B.W., Craighead W.E., Franco A.R., Craddock R.C., Mayberg H.S. (2013). Toward a neuroimaging treatment selection biomarker for major depressive disorder. JAMA Psychiatry.

