# Supplementary material for: Multiple Pre-Treatment miRNAs Levels in Untreated Major Depressive Disorder Patients Predict Early Response to Antidepressants and Interact with Key Pathways
Source: Int J Mol Sci. 2022 Mar 31;23(7):3873. doi: 10.3390/ijms23073873 (PMC8999364; doi:10.3390/ijms23073873)
Supplement: Supplementary file 1 [file ijms-23-03873-s001.zip › ijms-1643575-supplementary.pdf]

Supplementary Table S1. A total of 228 miRNAs significantly correlated with the changes in HAM-D score for SSRI at week 2 after FDR correction.

has-miRNA	coefficient	p-value	Adjusted p-value (FDR)	has-miRNA	coefficient	p-value	Adjusted p-value (FDR)	has-miRNA	coefficient	p-value	Adjusted p-value (FDR)
**miR-483-5p**	2.495	7.90E-05	0.0436	**miR-4726-5p**	2.923	0.0013	0.0183	**miR-4751**	2.602	0.0038	0.0271
**miR-3151-5p**	3.510	0.0001	0.0317	**miR-937-5p**	2.666	0.0013	0.0185	**miR-6870-5p**	2.070	0.0038	0.0268
**miR-7109-5p**	2.848	0.0001	0.0266	**miR-4294**	2.097	0.0013	0.0181	**miR-6731-3p**	4.188	0.0038	0.0266
**miR-6807-5p**	2.381	0.0002	0.0225	**miR-4769-3p**	2.648	0.0015	0.0198	**miR-6880-5p**	1.967	0.0039	0.0268
**miR-30c-1-3p**	2.138	0.0003	0.0276	**miR-6845-5p**	3.005	0.0016	0.0205	**miR-3135b**	2.552	0.0039	0.0265
**miR-6769a-3p**	3.474	0.0003	0.0234	**miR-6127**	2.389	0.0016	0.0201	**miR-6825-5p**	1.585	0.0040	0.0266
**miR-7111-3p**	3.241	0.0003	0.0232	**miR-197-3p**	2.742	0.0016	0.0198	**miR-4463**	2.856	0.0040	0.0265
**miR-6796-3p**	2.836	0.0003	0.0211	**miR-1281**	2.741	0.0017	0.0198	**miR-7114-5p**	1.637	0.0041	0.0269
**miR-1249-5p**	2.664	0.0003	0.0188	**miR-874-5p**	2.214	0.0018	0.0212	**miR-6824-5p**	2.167	0.0042	0.0274
**miR-4534**	2.329	0.0003	0.0170	**miR-6861-3p**	2.541	0.0018	0.0213	**miR-4749-3p**	2.409	0.0043	0.0276
**miR-4646-3p**	3.164	0.0003	0.0161	**miR-7107-5p**	2.371	0.0019	0.0211	**miR-6779-5p**	2.519	0.0044	0.0281
**miR-642b-3p**	2.659	0.0004	0.0172	**miR-6889-5p**	2.135	0.0019	0.0212	**miR-328-5p**	2.798	0.0045	0.0280
**miR-7111-5p**	2.765	0.0004	0.0160	**miR-1229-5p**	2.691	0.0020	0.0220	**miR-557**	2.425	0.0045	0.0277
**miR-1202**	2.514	0.0004	0.0153	**miR-6829-5p**	2.981	0.0021	0.0225	**miR-4783-3p**	2.604	0.0046	0.0282
**miR-4526**	3.096	0.0004	0.0148	**miR-6891-5p**	2.360	0.0022	0.0230	**miR-4723-3p**	1.991	0.0047	0.0283
**miR-193b-3p**	3.588	0.0005	0.0175	**miR-92a-2-5p**	1.797	0.0022	0.0230	**miR-4259**	2.583	0.0048	0.0286
**miR-7112-5p**	2.446	0.0006	0.0186	**miR-671-5p**	1.976	0.0023	0.0230	**miR-3162-5p**	2.479	0.0048	0.0284
**miR-6774-5p**	2.606	0.0006	0.0177	**miR-3679-3p**	2.464	0.0023	0.0229	**miR-4417**	2.533	0.0050	0.0294
**miR-642a-3p**	2.116	0.0006	0.0176	**miR-5196-5p**	2.537	0.0024	0.0233	**miR-5090**	2.479	0.0051	0.0296
**miR-6510-5p**	2.510	0.0007	0.0181	**miR-4479**	3.048	0.0024	0.0232	**miR-4725-5p**	2.719	0.0053	0.0303
**miR-6891-3p**	3.405	0.0007	0.0176	**miR-3141**	2.109	0.0025	0.0230	**miR-6768-5p**	1.747	0.0053	0.0300
**miR-7150**	2.328	0.0007	0.0181	**miR-6808-5p**	2.117	0.0025	0.0230	**miR-8089**	2.655	0.0053	0.0298
**miR-3614-5p**	2.691	0.0007	0.0176	**miR-2861**	4.272	0.0025	0.0229	**miR-3154**	1.659	0.0053	0.0295
**miR-4758-3p**	2.784	0.0008	0.0174	**miR-4687-5p**	2.572	0.0025	0.0227	**miR-6786-5p**	4.067	0.0055	0.0305
**miR-7155-5p**	2.668	0.0008	0.0170	**miR-6085**	2.660	0.0026	0.0229	**miR-658**	1.832	0.0057	0.0312

**miR-6124**	2.337	0.0008	0.0168	**miR-1275**	2.472	0.0027	0.0233	**miR-1233-5p**	2.322	0.0058	0.0316
**miR-6769b-5p**	2.714	0.0008	0.0171	**miR-675-5p**	2.019	0.0028	0.0237	**miR-6782-5p**	2.515	0.0059	0.0317
**miR-1273g-3p**	1.903	0.0009	0.0178	**miR-4687-3p**	2.589	0.0029	0.0239	**miR-3187-3p**	1.492	0.0060	0.0319
**miR-3158-5p**	2.231	0.0010	0.0182	**miR-6781-5p**	2.994	0.0029	0.0243	**miR-4689**	2.360	0.0064	0.0334
**miR-6738-5p**	3.034	0.0010	0.0181	**miR-1913**	2.632	0.0029	0.0239	**miR-6775-5p**	2.339	0.0067	0.0349
**miR-4723-5p**	1.987	0.0010	0.0184	**miR-6727-3p**	2.472	0.0030	0.0242	**miR-3197**	2.672	0.0069	0.0357
**miR-652-5p**	2.087	0.0010	0.0179	**miR-6797-3p**	2.389	0.0031	0.0247	**miR-3196**	4.162	0.0070	0.0358
**miR-6785-3p**	4.040	0.0011	0.0192	**miR-296-5p**	2.814	0.0031	0.0243	**miR-4728-3p**	2.185	0.0070	0.0355
**miR-6515-3p**	2.940	0.0012	0.0188	**miR-6845-3p**	2.814	0.0033	0.0255	**miR-6716-5p**	2.347	0.0071	0.0359
**miR-625-3p**	4.040	0.0012	0.0184	**miR-1343-5p**	2.584	0.0034	0.0255	**miR-532-3p**	2.731	0.0072	0.0359
**miR-6766-3p**	2.602	0.0012	0.0185	**miR-6894-3p**	2.026	0.0034	0.0253	**miR-6762-5p**	2.696	0.0072	0.0356
**miR-4739**	3.057	0.0012	0.0180	**miR-371a-5p**	2.635	0.0035	0.0257	**miR-4656**	2.146	0.0074	0.0361
**miR-4731-5p**	3.231	0.0013	0.0183	**miR-6799-5p**	2.109	0.0037	0.0268	**miR-6830-5p**	3.314	0.0076	0.0368
**miR-6816-5p**	3.085	0.0079	0.0379	**miR-6087**	3.338	0.0116	0.0418	**miR-6865-3p**	2.053	0.0159	0.0459
**miR-6749-5p**	2.485	0.0079	0.0377	**miR-6819-5p**	2.584	0.0117	0.0418	**miR-6885-3p**	2.078	0.0159	0.0457
**miR-7845-5p**	2.275	0.0081	0.0381	**miR-4530**	1.736	0.0117	0.0417	**miR-4749-5p**	2.063	0.0161	0.0460
**miR-6757-5p**	2.081	0.0081	0.0380	**miR-4433b-3p**	2.343	0.0120	0.0423	**miR-4327**	1.907	0.0161	0.0459
**miR-6797-5p**	2.479	0.0081	0.0377	**miR-6846-5p**	1.975	0.0120	0.0422	**miR-8071**	1.559	0.0163	0.0463
**miR-6879-5p**	2.274	0.0081	0.0374	**miR-6877-5p**	2.464	0.0121	0.0424	**miR-4701-5p**	2.787	0.0165	0.0465
**miR-6859-3p**	2.566	0.0081	0.0371	**miR-135a-3p**	2.071	0.0123	0.0427	**miR-4673**	2.080	0.0166	0.0466
**miR-6752-5p**	2.841	0.0082	0.0373	**miR-4507**	1.868	0.0124	0.0427	**miR-4763-5p**	1.812	0.0167	0.0466
**miR-6858-3p**	2.417	0.0084	0.0378	**miR-3180**	2.322	0.0124	0.0424	**miR-6813-3p**	2.279	0.0173	0.0479
**miR-4485-5p**	1.345	0.0085	0.0381	**miR-874-3p**	2.049	0.0125	0.0427	**miR-6769a-5p**	1.973	0.0176	0.0485
**miR-1249-3p**	2.795	0.0086	0.0379	**miR-3131**	2.007	0.0127	0.0432	**miR-548q**	1.668	0.0176	0.0483
**miR-760**	2.132	0.0087	0.0379	**miR-3195**	2.195	0.0128	0.0429	**miR-4685-5p**	2.596	0.0176	0.0481
**miR-6792-5p**	2.524	0.0087	0.0379	**miR-6805-5p**	2.802	0.0129	0.0430	**miR-3652**	1.721	0.0176	0.0479
**miR-6842-5p**	1.600	0.0087	0.0376	**miR-6860**	1.931	0.0130	0.0433	**miR-4443**	1.424	0.0178	0.0481
**miR-1228-5p**	3.208	0.0088	0.0376	**miR-6724-5p**	2.303	0.0132	0.0435	**miR-4758-5p**	2.379	0.0178	0.0479
**miR-3147**	2.260	0.0092	0.0390	**miR-6875-5p**	2.314	0.0132	0.0433	**miR-3122**	2.066	0.0178	0.0477

**miR-5572**	2.286	0.0093	0.0390	**miR-5698**	1.852	0.0132	0.0431	**miR-6812-5p**	2.044	0.0183	0.0487
**miR-6880-3p**	2.196	0.0093	0.0390	**miR-6810-3p**	2.124	0.0134	0.0435	**miR-3648**	2.965	0.0184	0.0489
**miR-6786-3p**	2.188	0.0094	0.0389	**miR-6813-5p**	2.228	0.0135	0.0435	**miR-4731-3p**	2.051	0.0186	0.0491
**miR-4750-5p**	1.518	0.0097	0.0398	**miR-6821-5p**	2.054	0.0135	0.0434	**miR-2116-3p**	2.121	0.0186	0.0489
**miR-4271**	1.940	0.0098	0.0401	**miR-1237-5p**	3.476	0.0135	0.0432	**miR-92b-5p**	1.972	0.0188	0.0493
**miR-575**	1.422	0.0098	0.0400	**miR-4433a-5p**	2.190	0.0138	0.0438	**miR-1237-3p**	1.640	0.0189	0.0491
**miR-4462**	2.103	0.0099	0.0397	**miR-665**	1.839	0.0139	0.0440	**miR-4707-5p**	1.927	0.0191	0.0494
**miR-187-5p**	1.553	0.0099	0.0397	**miR-6893-3p**	1.625	0.0140	0.0439	**miR-4632-5p**	1.715	0.0191	0.0493
**miR-6877-3p**	2.019	0.0100	0.0398	**miR-6076**	1.590	0.0140	0.0438	**miR-7847-3p**	2.132	0.0191	0.0491
**miR-4728-5p**	2.579	0.0100	0.0395	**miR-6765-3p**	1.376	0.0141	0.0437	**miR-6840-3p**	2.235	0.0193	0.0493
**miR-1228-3p**	2.624	0.0100	0.0393	**miR-4484**	1.768	0.0142	0.0439	**miR-3928-3p**	1.947	0.0194	0.0494
**miR-6875-3p**	2.599	0.0103	0.0401	**miR-550a-5p**	1.921	0.0143	0.0438	**miR-1914-3p**	2.302	0.0195	0.0494
**miR-1224-3p**	2.154	0.0104	0.0402	**miR-6800-3p**	2.350	0.0144	0.0438	**miR-197-5p**	2.050	0.0196	0.0494
**miR-4279**	2.022	0.0109	0.0416	**miR-718**	2.307	0.0145	0.0441	**miR-6801-3p**	2.218	0.0197	0.0494
**miR-6729-3p**	2.113	0.0109	0.0417	**miR-6862-3p**	1.666	0.0148	0.0448	**miR-3180-3p**	2.286	0.0197	0.0492
**miR-4763-3p**	1.893	0.0110	0.0414	**miR-6827-5p**	2.062	0.0152	0.0455	**miR-6858-5p**	2.107	0.0197	0.0490
**miR-7113-3p**	2.019	0.0110	0.0413	**miR-1225-5p**	1.959	0.0152	0.0454	**miR-1236-3p**	2.211	0.0198	0.0491
**miR-1227-5p**	2.268	0.0110	0.0410	**miR-2110**	2.289	0.0152	0.0451	**miR-7108-5p**	2.042	0.0202	0.0498
**miR-4459**	1.632	0.0111	0.0410	**miR-4695-3p**	1.605	0.0155	0.0458	**miR-4800-5p**	1.700	0.0204	0.0500
**miR-4449**	2.224	0.0114	0.0418	**miR-6787-5p**	2.243	0.0155	0.0456	**miR-6851-5p**	1.997	0.0204	0.0498
**miR-6803-5p**	2.620	0.0114	0.0418	**miR-371b-5p**	1.913	0.0156	0.0455	**miR-3185**	1.688	0.0205	0.0498
**miR-4651**	2.496	0.0115	0.0418	**miR-6867-3p**	2.123	0.0156	0.0454	**miR-4741**	1.701	0.0206	0.0499
